# Low Intensity Thermal Stimulation to Enhance Early Osteointegration in Implants: A Preclinical Study in Rabbits

**DOI:** 10.1002/cre2.70223

**Published:** 2025-09-19

**Authors:** Zhu Xiufeng, Wang Miao, Zhou Huixia, Xu Boya, Chang Xiaofeng, He Longlong

**Affiliations:** ^1^ Key Laboratory of Shaanxi Province for Craniofacial Precision Medicine Research, College of Stomatology Xi'an Jiaotong University Xi'an China; ^2^ Department of Implant Dentistry College of Stomatology, Xi'an Jiaotong University Xi'an China

**Keywords:** dental implant, implant stability, low‐intensity thermal stimulation, osseointegration, reverse torque

## Abstract

**Objectives:**

This study is the first to integrate 3D finite element modeling, in vitro validation, and preclinical animal experiments to determine the efficacy of low‐intensity thermal stimulation (LITS) in enhancing dental implant osseointegration. The study seeks to provide experimental evidence for applying thermal stimulation as a possible approach to enhance osseointegration.

**Material and Methods:**

A 3D finite element implant‐femur model and in vitro implant‐bone system were developed to simulate heat distribution. LITS conditions (50°C/5 s) were validated to avoid exceeding the osteoblast safety threshold (47°C). Eighteen rabbits received femoral implants divided into: control (no heating), T1 (single 50°C/5 s heating cycle), and T2 (three cycles). Outcomes included implant stability (IST), reverse torque, bone volume fraction (BV/TV), and histomorphometric osseointegration rate at 6 weeks.

**Results:**

Finite element and in vitro analyses confirmed 50°C/5 s as the optimal protocol, maintaining implant surface temperatures ≤ 46.3°C and complete thermal recovery within 1 min. T1 significantly increased in vivo rabbit model reverse torque (*p* < 0.05) and BV/TV (*p* < 0.05), while T2 showed no BV/TV improvement. Both T1 and T2 exhibited higher osseointegration rates (*p* < 0.05). Implant stability (IST) remained unchanged across groups (*p* > 0.05).

**Conclusion:**

LITS at 50°C/5 s safely enhances early osseointegration in rabbits, increasing biomechanical anchorage and peri‐implant bone formation. This study provides preliminary experimental evidence for the potential of thermal application in enhancing implant osseointegration.

## 1 Introduction

Dental implant restoration has become the gold standard for replacing missing teeth, with long‐term success hinging on stable osseointegration – a direct structural and functional connection between bone and implant (Bosshardt et al. 2017; Albrektsson et al. 1981; Howe et al. 2019). Enhancing osseointegration, reducing healing time, and improving its quality are key research areas in the field of oral implantology. The patient's bone response ability plays a vital role in this biological process after implant placement, closely associated with systemic factors such as smoking, diabetes, and osteoporosis, negatively impacting bone‐implant contact and long‐term implant retention (Hasegawa et al. 2008; Raikin et al. 1998; Chen et al. 2013). While surface modifications and bioactive coatings aim to enhance osseointegration, these approaches often involve complex manufacturing or high costs, limiting accessibility (de Almeida et al. 2017; Liu et al. 2022). Thus, developing noninvasive, cost‐effective strategies to accelerate bone healing remains a critical unmet need.

Low‐intensity thermal stimulation (LITS) has emerged as a promising adjuvant therapy for bone regeneration due to its ability to modulate cellular behavior. Recent studies demonstrate that controlled hyperthermia (40°C–45°C) activates heat shock proteins (HSP70), stimulating osteoblast differentiation and angiogenesis while suppressing pro‐inflammatory cytokines like IL‐6 and TNF‐α (Weinberger et al. 1990; Carter et al. 1998; Ikenaga et al. 1994; Palanisamy et al. 2022; Castelló et al. 2022). The study identified 47°C as the critical threshold for bone necrosis, emphasizing the precision required in thermal dosing (Eriksson and Albrektsson 1983; Kniha et al. 2020). Studies implied the potential impacts of thermal treatment on bone dynamics. When LITS is applied directly to specific sites, the temperature directly influences various cellular components, including osteoblasts (Brookes et al. 1970). Cellular experiments have demonstrated that rat calvarial osteoblasts exposed to temperatures between 42°C and 45°C for 1 min can reassemble damaged cell skeletons, while irreversible damage occurs at 48°C (Li et al. 1999; Kniha et al. 2024). Moreover, LITS holds the potential for accelerating local bone tissue formation by promoting the proliferation and differentiation of osteoblasts and mesenchymal stem cells (Dolan et al. 2012; Shui and Scutt 2001). Yet its application in dental implants remains underexplored, with only one report using the thermal application in implant removal (Kniha et al. 2024). Current gaps include undefined optimal thermal parameters and a lack of integrated biomechanical validation.

We hypothesize that LITIS delivered at carefully calibrated parameters (≤ 47°C) enhances peri‐implant bone remodeling by synergistically activating osteogenic signaling pathways while avoiding thermal damage. Using 3D finite element analysis, we first defined a safe thermal protocol, which was subsequently validated in vitro and tested in a rabbit femoral implant model. Our findings provide critical insights into LITS‐mediated osseointegration, offering a potential solution to improve implant outcomes in high‐risk patient populations.

## 2 Materials and Methods

### 2.1 Simulation of Heat Conduction Using 3D Finite Element Method

The process of constructing the implant‐bone model involved several stages, including scanning, segmentation, and assembly (Figure S1). Microcomputed tomography (Micro‐CT, Cheetah, Comet Yxlon, Germany) scans were performed on the implant to obtain a digital model, which was then exported in DICOM format (Digital Imaging and Communications in Medicine). Mimics Medical 21.0 software (Materialise, Leuven, Belgium) was utilized to create the model by identifying and segmenting the implant using the mask tool (Feng et al. 2018). The resulting STL format file was imported into Geomagic Wrap 2017 software (3D Systems, USA) for further refinement. The same procedure was followed to create a model of the rabbit femur. The rabbit femur and implant models were assembled in Solidworks 2018 (Dassault Systemes, France) to produce the femur‐implant model (Naguib et al. 2023). The simulation utilized ANSYS Workbench 2020 software (ANSYS Inc., Canonsburg, Pennsylvania, USA), assuming isotropic materials for the model parameters (Haseeb et al. 2022) (detailed setting is shown in Table S1). Model parameters were configured to simulate different heating situations, and the temperature distribution was evaluated at designated points on the implant surface. The heating was loaded by designating the heating surface as the bottom of the abutment screw (Figure S1). The heat transfer coefficient was set at 500 J/(m^2^·s·K), and the ambient temperature was set to 38°C, which is the average body temperature of a rabbit (Edis et al. 2022).

### 2.2 Determination of Titanium Implant Temperature Conduction in In Vitro Tissue

For the in vitro experiment, Trausim implants (4.1 mm × 10 mm; China) were placed into fresh pig ribs to create an in vitro implant‐bone model, which was put in a 38°C water bath before the experiment. Electric heating device (DBS, Germany) was used to heat the bottom of the abutment screw, and implant surface temperatures were measured at three sites (P1, P2, and P3) using a multi‐channel thermometer (Changzhou Jin Yi Electronic Technology Co. Ltd.) (Figure S2). The computer program recorded the temperature changes with a recording interval of 1 s.

### 2.3 Application LITS on the Osseointegrated Implant in Rabbit Model

Eighteen male New Zealand white rabbits (mean age 6 months, mean weight 2.9 kg) were individually housed under clean, dry, and well‐ventilated conditions. This study was approved by the Ethics Committee of the College of Stomatology, Xi'an Jiaotong University, Xi'an, China (Ethic approval No. [2016]020). All animal experiments were conducted in strict compliance with the ARRIVE guidelines to ensure appropriate animal welfare. The rabbits were randomly assigned and divided into three groups (N = 18): control group (Group Ctr), Group T1 (50°C heating for 5 s once), and Group T2 (50°C heating for 5 s three times, with a 1‐min interval between each cycle). Specimens were examined using cone beam computed tomography (CBCT), and three‐dimensional data on the femur were obtained. All operations and measurements were performed under general anesthesia using 1 mg/kg propofol via venae auricularis and maintained with 1%‐3% isoflurane mixed with pure oxygen via a face mask (Figure S3). Local infiltration anesthesia was performed with 2% lidocaine. Trausim implants (4.1 mm × 10 mm) were placed in the femur with an insertion torque greater than 25 N·cm. The implant stability quotient (ISQ) value was measured by Anycheck (Neobiotech, Korea) (J. Lee et al. 2020; D.H. Lee 2020). Cover screws were then used to cover the implants. After surgery, penicillin sodium (8 × 10^5^ U/kg) was administered intramuscularly for 3 days to prevent infection. Three weeks after implant placement, the implant cover screw was removed, applying thermal stimulation to the implant according to the group setting.

### 2.4 Data Collection and Statistical Analysis

After applying the thermal stimulation according to group setting, the rabbits were euthanized at 6 weeks, and the femurs were collected for further data collection: (1) Stability testing of implants: implant stability test (IST) values were measured using Anycheck in four directions of the implant at 3 weeks after implant placement and before the heating application, and at 6 weeks after implant placement (Figure S4A). Three measurements were taken in each direction and the average of the four directions IST values was taken as the IST measurement result of the implant. (2) Reverse torque of implants: The DS2‐50N digital push‐pull force gauge (Dongguan Zhiqu Precision Instrument Co. Ltd., China) was used to measure the maximum pulling force during the reverse rotation process of the implant by applying force slowly until the implant was loosened in the opposite direction. The calculation method for the reverse torque experiment results of the implants is as follows: reverse torque (N·cm) = digital push‐pull force gauge display value (N) × torque wrench length (cm) (Figure S4B–C). (3) Morphological analysis of bone around implants: The femoral bone specimens were scanned by Micro‐CT (Comet Yxlon; Y. Cheetah, Germany). After 3D reconstruction by VG studio 3.0 software, the bone volume fraction (Bone volume/Total volume, BV/TV) in the neck and root regions of the implant was calculated using threshold selection after defining the region of interest (ROI) as a cylindrical area 100‐300 μm away from the surface of the implant (Figure S4D). (4) Analysis of implant osseointegration rate: The femoral bone specimen with the implant was fixed with 4% paraformaldehyde solution for 48 h and then dehydrated and embedded in Technovit 7200 resin. The specimen was cut into 200 μm sections and stained with methylene blue and acid fuchsin. The degree of implant‐bone integration was observed and calculated using BIOQUANT OSTEO 2020 software. (5) Data Analysis: IBM SPSS Statistics 26.0 software was used for data analysis. The mean ± standard deviation was used to represent all numerical results. One‐way ANOVA was used for data with normal distribution, and the least significant difference (LSD) test was used for post‐hoc analysis of differences.

## 3 Results

### 3.1 Optimal LITS Conditions Determined Using 3D Finite Element Method

**Figure 1:**
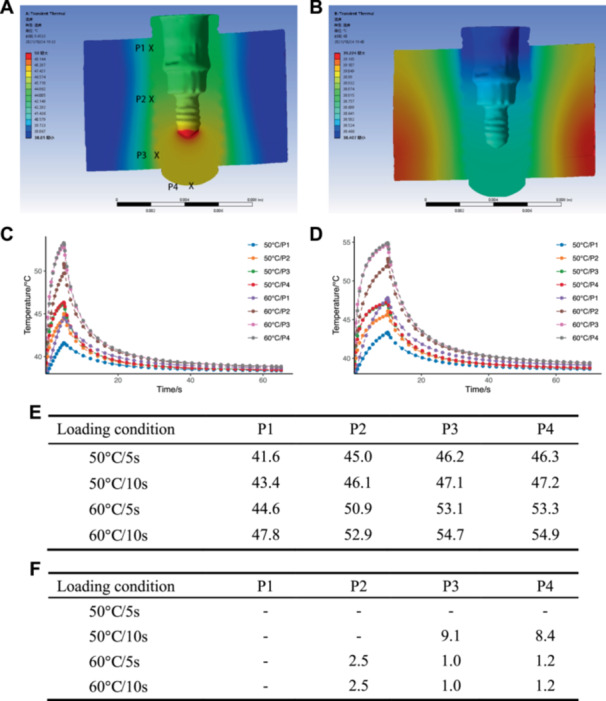
Temperature field distribution in the model after 10 s of heating at 50°C and 1 min of cooling. (A) Temperature distribution around the implant after 5 s of heating. (B) Temperature distribution around the implant after 1 min of cooling. (C) Temperature variations of various P‐points during a 5‐second heating load. (D) Temperature variations of various P‐points during a 10‐second heating load. (E) Maximum temperatures (°C) of various P‐points on the implant's surface in the model. (F) Time (s) taken for various P‐points in the model to reach 47°C.

The optimal heating conditions were determined using the 3D Finite Element Method, and the temperature field changes inside the implant and surrounding tissues were analyzed in ANSYS Workbench. The temperature distribution during the heating and heat dissipation process was observed to be uniform around the implant, gradually decreasing as the distance from the implant surface increased (Figure 1A,B). The temperature changes around the implant under different loading conditions are shown in Figure 1C,D, and when the load is applied for 5 s, the implant surface temperature rises rapidly. At the same time, the temperature at the implant root tip 1/2 is slightly higher than that at the implant neck 1/2, with the temperatures at P3 and P4 rising relatively equally and faster than those at P1 and P2 (Figure 1C). The temperature at P1 rises the slowest, which is consistent with the temperature conduction characteristics of lower temperatures further from the heat source. Upon extending the load to 10 s, the temperature rise rate at P‐points gradually slows down as time increases (Figure 1D).

In this study, four points (P1, P2, P3, P4) were selected to represent the temperature changes at different positions on the implant surface. The maximum temperatures at each P‐point during the heat conduction process and the time to reach 47°C are shown in Figure 1E,F. When the load condition is 50°C/5 s, the maximum temperatures at P1, P2, P3, and P4 are 41.599°C, 45.014°C, 46.228°C, and 46.332°C, respectively, all not reaching 47°C, and recovering to close to 38°C within 1 min of stopping heating. The implant surface reaches 47°C at different times under the other load conditions. These LITS load conditions of 50°C/5 s are used as guidance for further study.

### 3.2 Characteristics of LITS Temperature Conduction in Titanium Implants In Vitro Tissue

**Figure 2:**
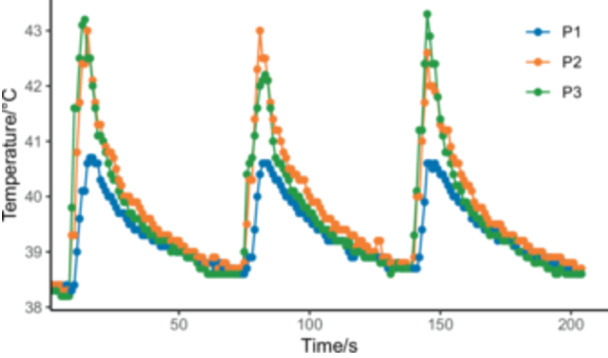
Temperature change chart of the implant's surface during a 50°C heating for 5 s and a 1‐min cooling cycle.

An in vitro bone model with implants was established to simulate thermal conduction in a physiological environment to validate the 3D finite element simulation results. The implant‐bone in vitro model was established with approximately 25 N·cm torque. The heating device was calibrated at 49.7 ± 0.8°C when the T12 intelligent multifunctional soldering station was set to 50°C. During the heating of the implant's central screw bottom, the temperatures at points P1, P2, and P3 were 40.6 ± 0.2°C, 43.0 ± 0.2°C, and 42.8 ± 0.5°C, respectively. After stopping the heating, the implant surface temperature returned to 38.7 ± 0.1°C within 1 min (Figure 2). It was confirmed that the temperature at all P‐points would not reach 47°C when the implant was heated at 50°C for 5 s in the actual in vitro process, and the temperature could return to ambient levels within 1 min.

The result showed that in the repeating heating cycle, heating at 50°C for 5 s with 1‐min intervals, the temperatures at all P‐points were also below 47°C, meeting the study requirements and providing operational guidance for further animal experiments. 50°C for 5 s was proposed as the intervention condition for the further animal experiments. Two experimental groups were set up: Group T1, with a cycle 5 s heating at 50°C; Group T2, with three cycles of 5 s heating at 50°C with a 1‐min interval between each cycle; and a non‐intervention blank control group.

### 3.3 Reverse Torque Increased After LITS Application on Titanium Implant

All 18 New Zealand White rabbits survived the surgery with stable vital signs and no deaths. They all exhibited good soft tissue healing, no signs of infection, and no severe adverse events such as fractures or deaths throughout the experiment. All implants showed no clinical mobility and achieved successful clinical osseointegration, with no signs of inflammation in soft or hard tissues or exudation.

The IST values at 3 and 6 weeks after implant placement are shown in Table 1. The measurement results showed that IST values > 65, which indicates all implants achieved good stability at 3 weeks after implant placement. The analysis showed no statistically significant difference in IST values between the three groups at 3 or 6 weeks (*p* > 0.05). The IST values at 6 weeks after implant placement were lower than those at 3 weeks, and the decrease was more pronounced in the treatment group compared to the control group. However, when the IST change values of each group were analyzed, there was no statistically significant difference.

**Table 1:** IST values of implants at 3 weeks and 6 weeks after implant placement.

Group	3 weeks (X ± SD) (*n*)	6 weeks (X ± SD) (*n*)	3 w–6 w (X ± SD) (*n*)
C	85.5 ± 2.5 (6)	83.7 ± 2.7 (6)	1.9 ± 4.2 (6)
T1	86.5 ± 1.0 (6)	81.4 ± 2.6 (6)	5.2 ± 2.7 (6)
T2	86.4 ± 0.6 (6)	81.3 ± 1.6 (6)	5.1 ± 1.5 (6)
*p*	0.508	0.171	0.130

*Note: p:* The statistical results showed that the IST values were normally distributed, and a one‐way ANOVA was used to describe the differences between the groups.

**Figure 3:**
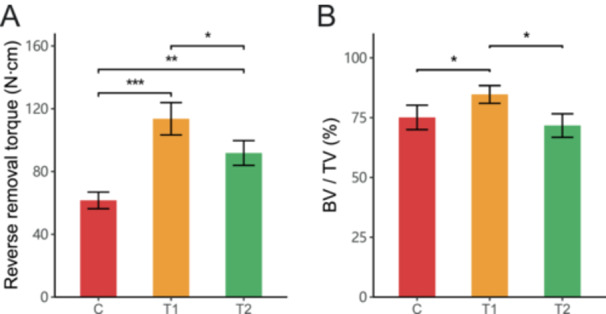
Reverse removal torque (N·cm, A) and Bone volume fraction (%, bone volume/total volume, BV/TV, B) in different groups (C, T1, T2). *p < 0.05, **p < 0.01, ***p < 0.001.

The reverse torque of the implant main body at the length of 7 cm was measured, as shown in Figure S4B,C. The reverse torque values of the implants at 6 weeks after implant placement are shown in Figure 3A. After the heating application, the reverse torque of the implants in the treatment group increased significantly compared to the control group. The reverse torque of the implants in Group T1 increased by 84.6%, and Group T2 increased by 49.1% compared to the control group (Control group: 61.6 ± 5.3 N·cm; T1: 113.6 ± 10.3 N·cm; T2: 91.8 ± 7.9 N·cm). The increase in Group T1 was more pronounced than in Group T2, with the reverse torque increase in Group T1 being 1.72 times that of Group T2. The results showed that the reverse torque differences between the three groups were statistically significant (*p* < 0.05).

### 3.4 Micro‐CT Analysis and Histomorphometry Showed a Higher Implant Osseointegration Rate After the LITS Application

After scanning the femur specimens with implants using Micro‐CT, a total of 1024 projections were obtained. The resolution in all directions was 19 microns after 3D reconstruction. The bone tissue in the ROI was selected after threshold segmentation, and the surrounding bone tissue of the implant was visually observed after rendering (Figure S4D). The BV/TV measurement in the ROI results at 6 weeks are shown in Figure 3B. After the heating intervention, the results showed that the bone volume fraction around the implant in Group T1 increased by 12.8% compared to the control group, while in Group T2, it decreased by 4.5% compared to the control group. The results showed that the reverse torque differences between the three groups were statistically significant (*p* < 0.05). The post hoc test using the LSD method showed no statistically significant difference between the control group and Group T2 (*p* > 0.05), and there were statistically significant differences between the control group and Group T1 (*p* < 0.05).

**Figure 4:**
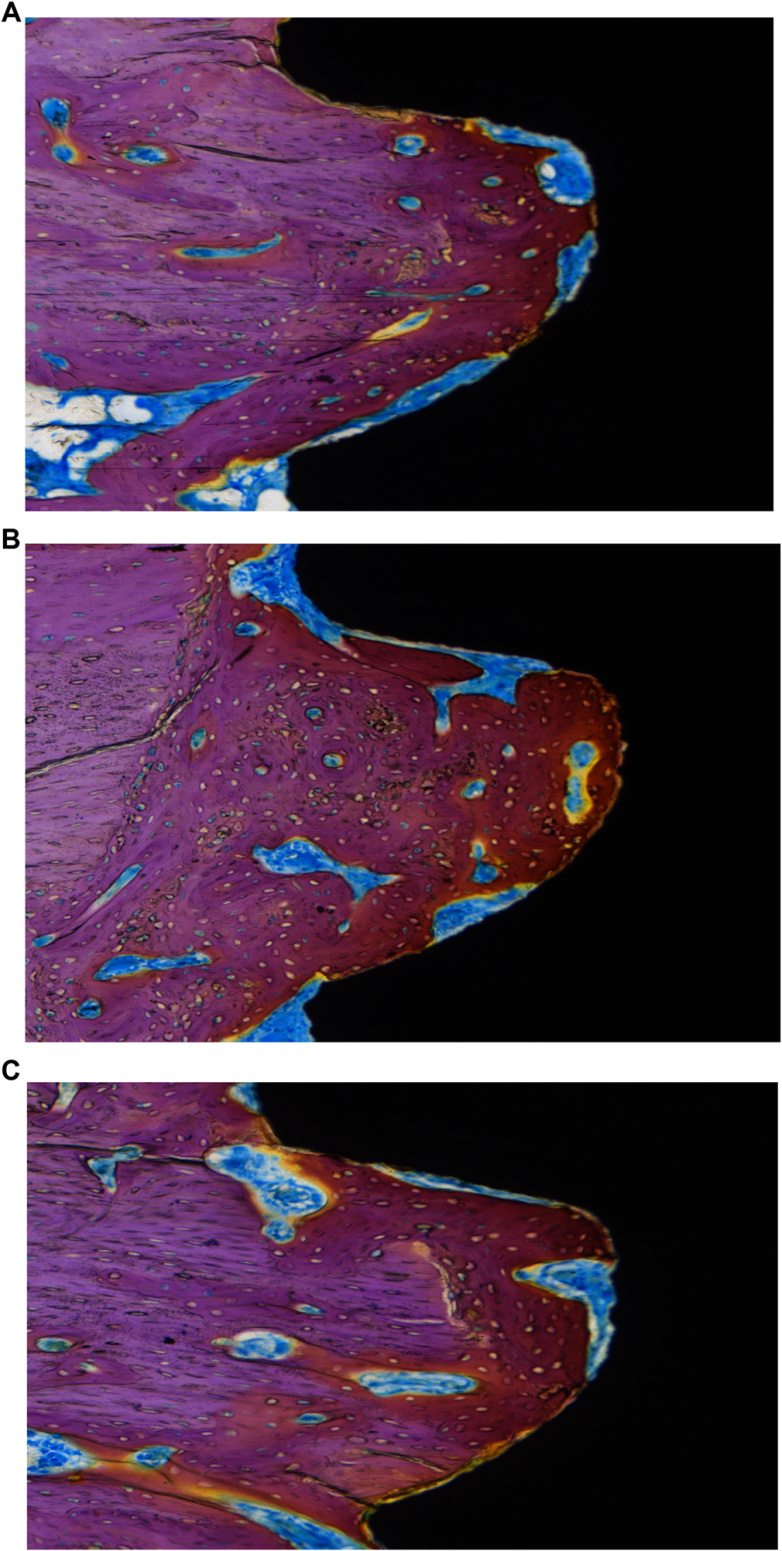
Histological section of implant‐bone interface. (A) C group. (B) T1 group. (C) T2 group. Newly formed bone appeared as a deep red color. The qualified method used for analysis involved staining the implant‐bone tissue section and placing it under the ASI imaging camera system for image capture at ×10 magnification. The images were imported into the BIOQUANT OSTEO 2020 software for observation and measurement of the implant‐bone integration rate, calculated as the length of direct contact between the implant and bone tissue divided by the total length of the implant edge multiplied by 100%.

Histological observations of the tissues surrounding the implant are shown in Figure 4. The implant sections showed good osseointegration, with direct contact between the implant surface and bone tissue. It can be seen that all three groups had a large number of osteoblasts and osteoclasts around the implant, indicating that active bone formation and remodeling were still occurring around the implant. In treatment groups T1 and T2, the newly formed bone was significantly more abundant around the implant than in control group (*p* < 0.05). The osseointegration rate measurement results showed that the treatment group had a significant increase in osseointegration rate compared to the control group (*p* < 0.05), with the osseointegration rate in Group T1 increased by 29.0% compared to the control group, while in Group T2, it increased by 22.6%.

## 4 Discussion

Recent literature reviews have indicated that LITS could potentially enhance bone tissue formation (Weinberger et al. 1990; Carter et al. 1998; Ikenaga et al. 1994; Palanisamy et al. 2022; Castelló et al. 2022; Brookes et al. 1970; Dolan et al. 2012; Shui and Scutt 2001; Porter et al. 2022). However, it is essential to find appropriate heating conditions, as temperatures above 47°C for 1 min can cause irreversible bone damage (Kniha et al. 2020; Li et al. 1999). This study aimed to find an accurate temperature setting and investigate the effects of thermal stimulation on implant osseointegration.

In a 3D finite‐element analysis, different implant surfaces were simulated for heating. The bottom surface of the implant's abutment screw channel was identified as the best for even and safe thermal distribution (Figure S1), keeping the temperature below 47°C. However, the 3D finite‐element analysis has limitations, like not being able to model heat loss at the contact point between the heating device and the surface, and the contact area affecting energy transfer.

In in‐vitro implant‐bone tissue, the actual temperatures around the implant were lower than simulated. Heat loss between the heating probe and the implant and the use of a water‐bath environment with a high specific heat capacity were the reasons (Vaupel and Piazena 2022). Still, the implant‐surface temperatures were below 47°C, which was suitable for further animal experiments. Implant stability and osseointegration are vital for long‐term dental implant success. This study evaluated them comprehensively using methods like implant stability testing (IST), reverse torque testing, micro‐CT analysis for bone volume fraction (BV/TV), and histological assessment of osseointegration. The IST results revealed that implant stability remained consistent across all treatment groups. However, this finding does not fully confirm the absence of adverse thermal effects on implant stability in the short term – nor can it completely rule out subtle early‐stage biological changes that do not manifest as detectable differences in stability measurements (J. Lee et al. 2020). Reverse torque testing, which measures the minimum torque to loosen the implant and represents osseointegration, showed that the treatment group had a significantly higher reverse torque than the control group. Group T1 had the highest, followed by T2, suggesting an ideal treatment condition between the control and T2 (Park et al. 2019; Johansson and Albrektsson 1987).

Micro‐CT was used to calculate BV/TV (Clark and Badea 2021; Song et al. 2021; Marquis et al. 2023). By setting the region of interest 100–300 microns from the implant surface to avoid the partial‐volume effect, it was found that heating at 50°C for 5 s (group T1) effectively promoted bone‐tissue deposition and growth around the implant, while heating three times continuously did not have a significant effect. The temperature conditions were in line with a recent study showing that heating a dental implant to 50°C for 1 min does not cause disintegration (Kniha et al. 2024).

Histological observation confirmed better bone‐implant contact in the treatment groups, indicating that short‐term heat stimulation can increase the osseointegration rate. A large amount of new bone was observed around the implants in the treatment group, which is consistent with the results of Leon et al.'s study (Leon et al. 1993). The reason may be that heat stimulation enhances the differentiation of osteoblast precursors and the deposition of new bone tissue on the surface (Dolan et al. 2012; Shui and Scutt 2001; Nørgaard et al. 2006). Moreover, though both group T1 and T2 promoted osseointegration regarding different perspectives, group T1 obtained better effects than group T2. The reason may be that when heat stimulation is applied to the implant, the surrounding tissue cells are sensitive to temperature changes. Mild heat stimulation can cause repairable damage to the cells and further enhance their resilience, resulting in better implant osseointegration in the heating group compared to the control group. This study initially hypothesized that three heating cycles might enhance osteogenic effects by sustaining HSP70 activation, but the weaker effect of T2 may be attributed to cumulative mild cellular stress: although each 50°C/5 s cycle alone does not exceed the 47°C safety threshold, repeated stimulation within short intervals (1 min) might disrupt the normal progression of osteoblast differentiation or induce excessive expression of mild inflammatory factors, thereby offsetting the positive effects of thermal stimulation. However, this mechanism remains speculative and requires further in vitro studies (e.g., cell culture experiments with repeated thermal stimulation) to verify.

The current study has some limitations, including using the rabbit femora, which differ from human jawbone in terms of microstructure and remodeling rate. Future studies should utilize animal models that better mimic human oral bone conditions (e.g., beagle dogs or minipig jaw models) to further verify the efficacy and safety of LITS before any clinical application is considered (Scarano et al. 2024). Additionally, the small sample size and short observation period warrant further research with larger sample sizes and extended observation periods to validate the findings. Future studies should extend the follow‐up period to at least 3–6 months to evaluate the long‐term stability of implants after LITS and whether the early enhanced osseointegration can be maintained over time. Although these methods have some limitations, the results collectively demonstrated that LITS could enhance osteoblast precursor differentiation and surface deposition of newly formed bone tissue, thereby improving the implant's removal torque and osseointegration rate.

In conclusion, this study is the first to demonstrate the positive effect of thermal stimulation on implant osseointegration using a combination of software simulations, in vitro tissues, and animal models. Our findings suggest that early short‐term thermal stimulation can enhance implant osseointegration, with the preliminary result obtained by heating the implant at 50°C for 5 s once. These findings provide a foundation for integrating LITS techniques and improving the implant osseointegration rate in animal models. However, it is important to recognize that the optimal conditions for thermal stimulation may vary based on factors such as implant size, patient characteristics, and device specifications. Therefore, further research is essential to address the limitations of this study and gain a deeper understanding of how to effectively apply LITS to enhance osseointegration in a clinical setting.

## 5 Conclusion

Integrating digital modeling simulation, in vitro experiments, and animal models provided a preliminary understanding of the effects of LITS on bone tissue and implant osseointegration. The results unequivocally demonstrated that LITS stimulates bone tissue deposition around the implant and improves osseointegration. These findings have significant implications for future research to determine this LITS's optimal device, timing, and parameters for this approach. By illuminating the positive effects of thermal stimulation on implant osseointegration, our study opens up possibilities for further investigations in this field.

## Author Contributions

Wang Miao contributed to conception and design, analysis and interpretation, drafted the manuscript, and critically revised the manuscript. Zhu Xiufeng contributed to conception and design, acquisition and interpretation, drafted the manuscript, and critically revised the manuscript. Xu Boya contributed to design, analysis and interpretation, drafted the manuscript, and critically revised the manuscript. Zhou Huixia contributed to conception, analysis and interpretation, and critically revised the manuscript. Chang Xiaofeng contributed to conception and design, analysis and interpretation, drafted the manuscript, and critically revised the manuscript. He Longlong contributed to conception and design, analysis and interpretation, drafted the manuscript, and critically revised the manuscript. All authors reviewed and approved the manuscript.

## Ethics Statement

This study was approved by ethics committee, College of Stomatology, Xi'an Jiaotong University, Xi'an, China (Ethic approval No. [2016]020).

## Conflicts of Interest

The authors declare no conflicts of interest.

## Supporting information


Supplementary Materials.

## Data Availability

The datasets used and/or analyzed in the current study are available from the corresponding author upon reasonable request.
