# Supplementary material for: Low Intensity Thermal Stimulation to Enhance Early Osteointegration in Implants: A Preclinical Study in Rabbits
Source: Clin Exp Dent Res. 2025 Sep 19;11(5):e70223. doi: 10.1002/cre2.70223 (PMC12446874; doi:10.1002/cre2.70223)
Supplement: Supplementary file 1 — Supplementary Materials. [file CRE2-11-e70223-s001.docx]

**Title of paper**

**Low-Intensity Thermal Stimulation to Enhance Early Osteointegration in Implants: A Preclinical Study in Rabbits**

Wang Miao1*, Zhu Xiufeng1*, Zhou Huixia1, Xu Boya1, Chang Xiaofeng1#, He Longlong1#

**Authors’ information:**

Key Laboratory of Shaanxi Province for Craniofacial Precision Medicine Research, College of Stomatology, Xi'an Jiaotong University, Xi’an, China.

* # Both the authors have contributed equally to the work

**Keywords:** dental implant; low-intensity thermal stimulation; osseointegration; implant stability; reverse torque


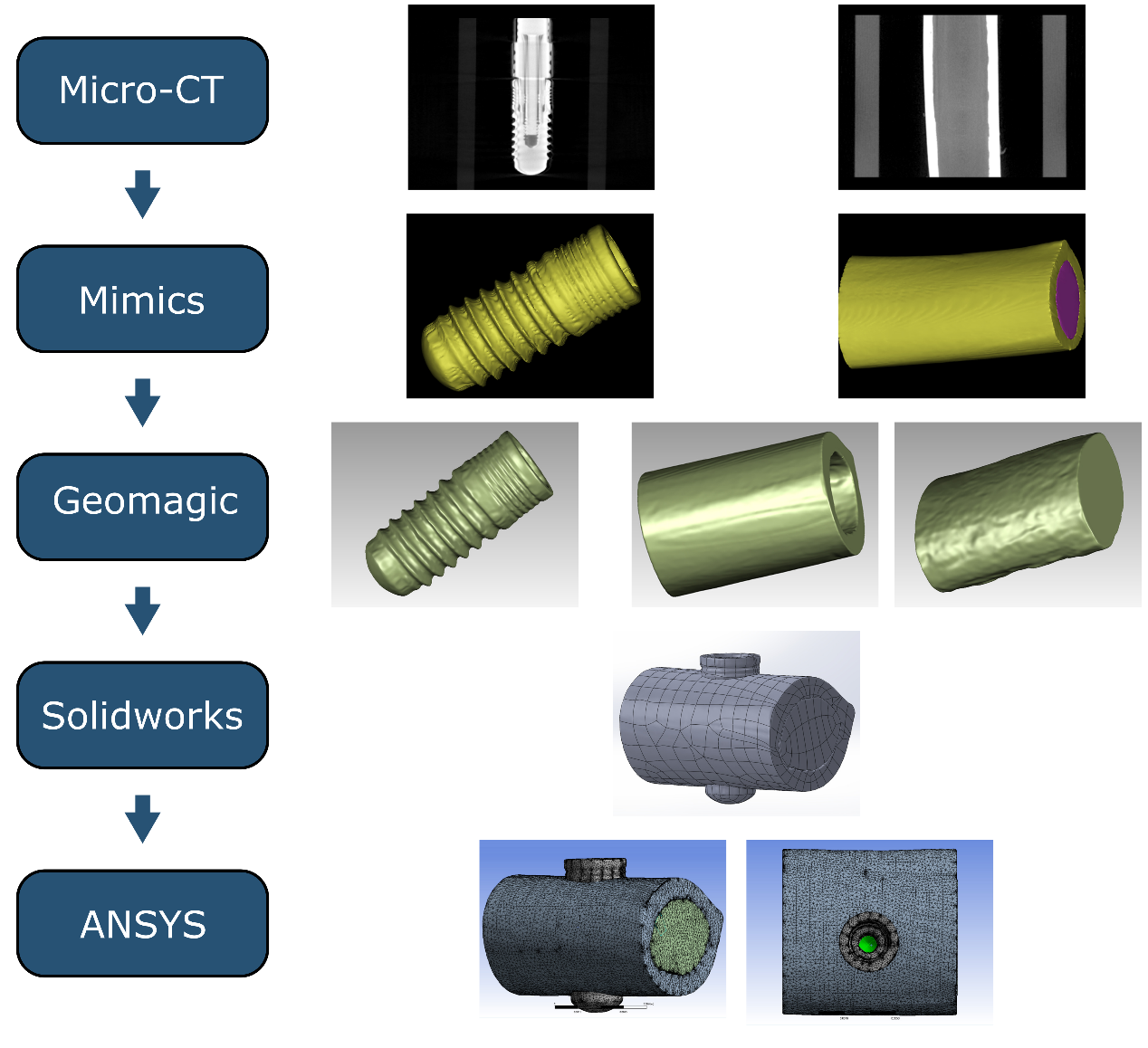


**Figure S1.** The workflow for constructing the 3D finite element analysis model.


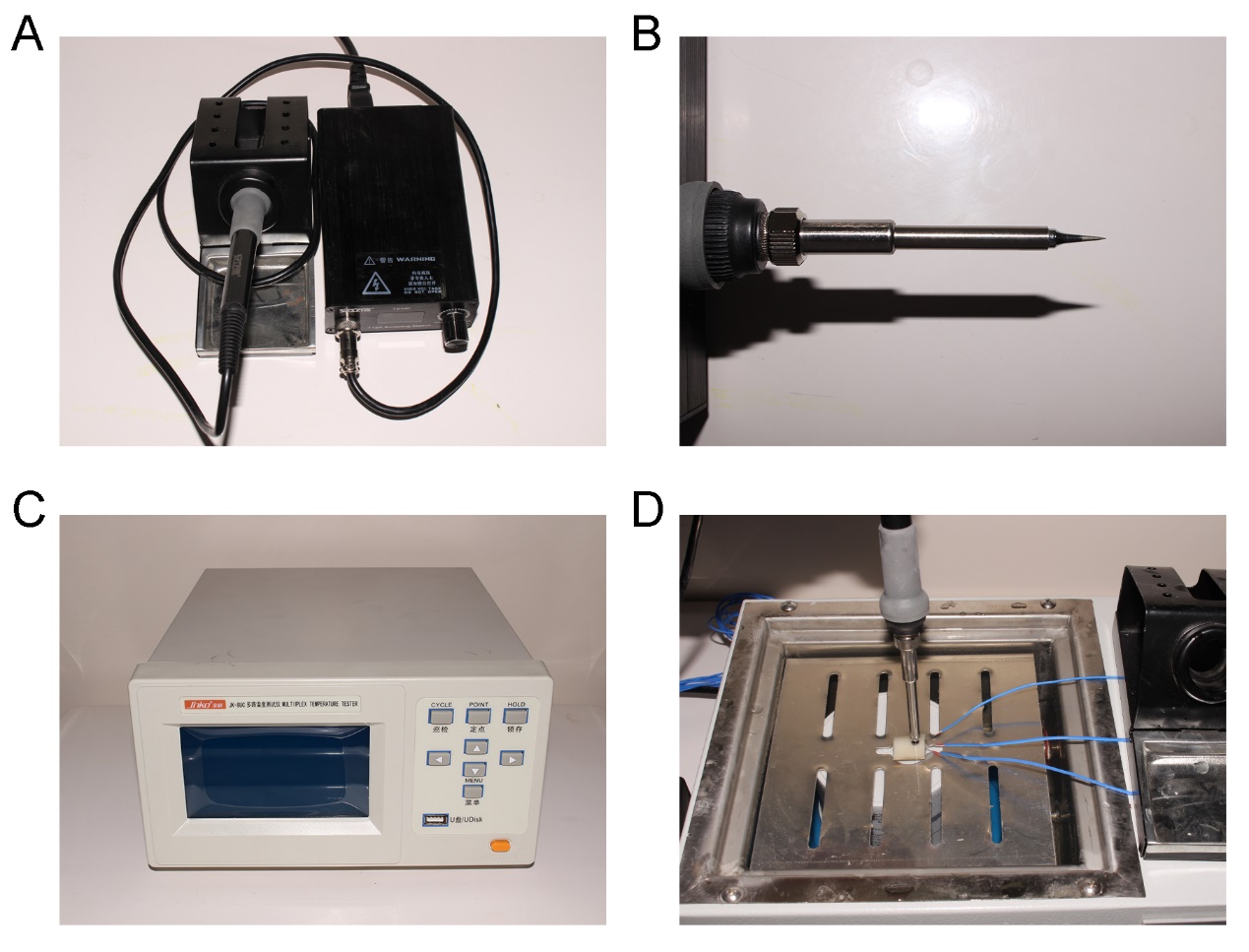


**Figure S2.** Thermal conductivity characteristics of titanium implant surface in vitro tissues. A. Atten ST-12 intelligent multi-function welding station. B. Atten ILS heating head. C. JK-8UC multi-channel thermometer. D. Temperature measurement of *in vitro* bone tissue.


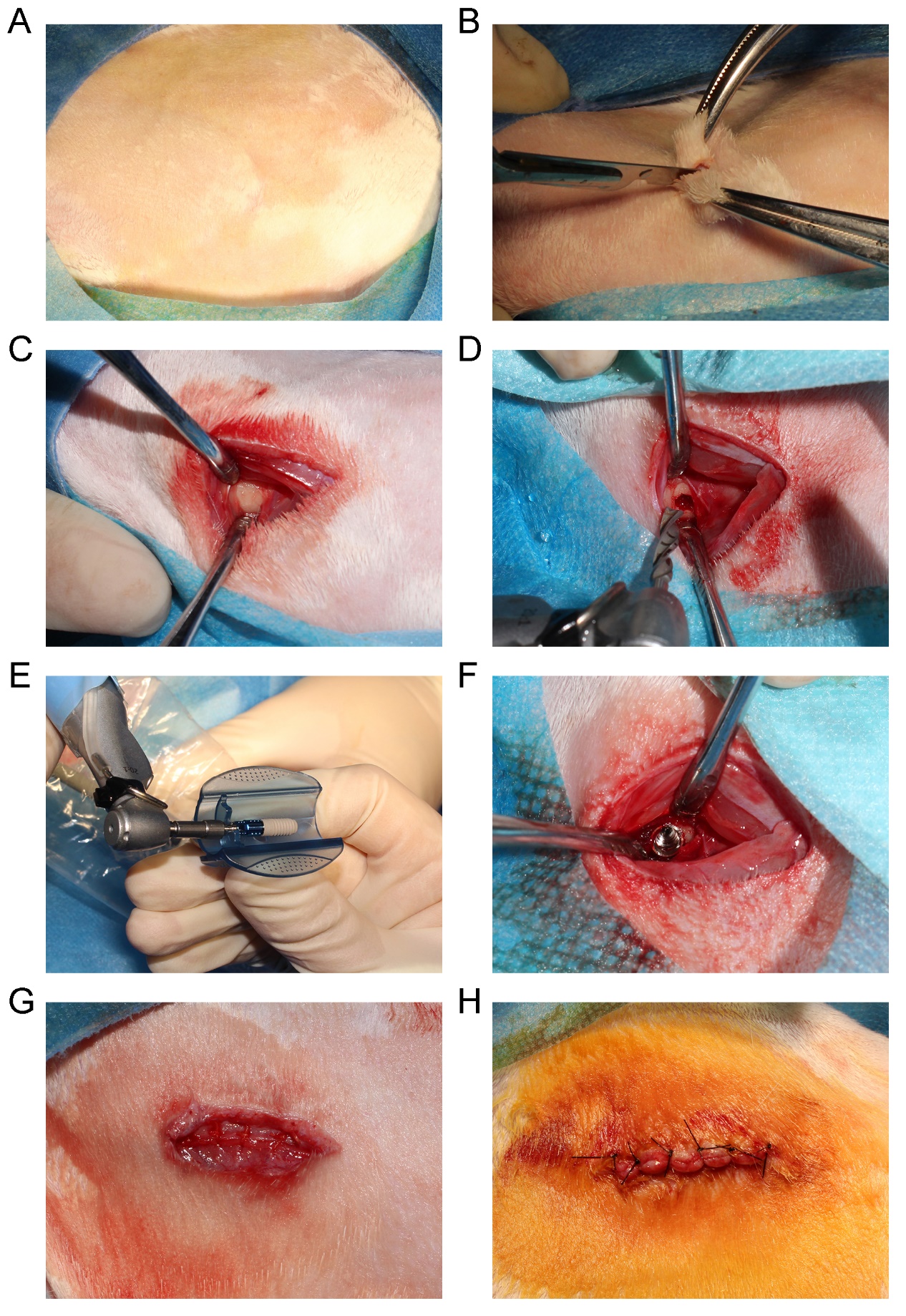


**Figure S3.** Surgical procedure for implant placement in the animal model involved the following steps: A. Preoperative skin disinfection and draping. B. Incision of the skin to expose the surgical site. C. Exposure of the femur. D. Preparation of the implantation socket. E. Implantation of the implant. F. Confirmation of the implant position. G. Closure of subcutaneous tissue layers using absorbable sutures. H. Closure of the skin using monofilament nylon sutures.


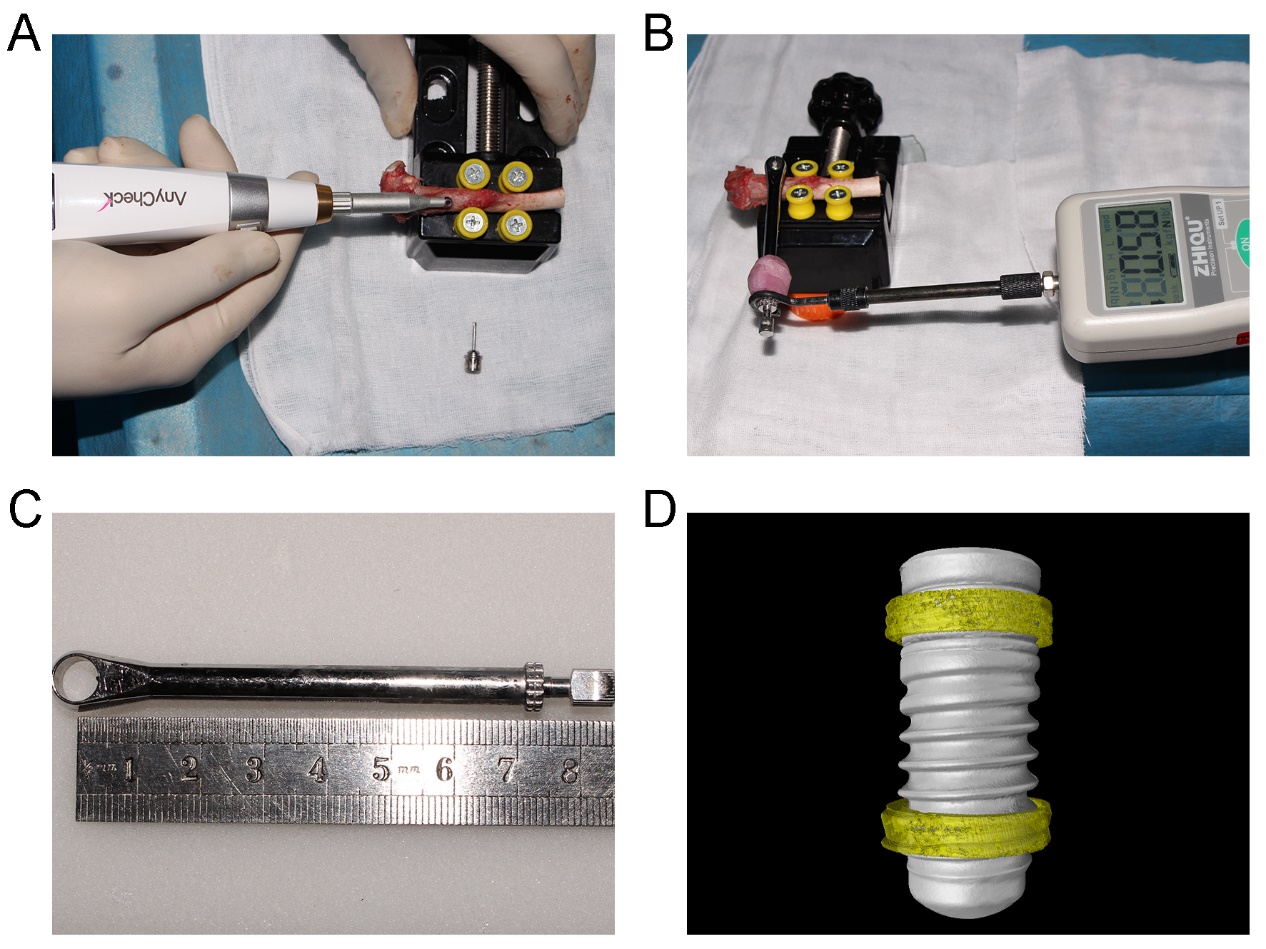


**Figure S4.** A. Implant Stability Test was tested using Anycheck. Reverse torque of implants. B-C. **B.** The DS2-50N digital push-pull force gauge was employed to measure the maximum pulling force. Force was applied gradually until the implant became loosened in the opposite direction. **C.** Torque wrench length. D. Schematic diagram of bone reconstructed by micro-CT.

| Table S1 Three finite element model parameters [1] | | | |
| --- | --- | --- | --- |
|  | Density  (kg/m^-3^) | Specific Heat Capacity (J/kg℃) | Thermal Conductivity (J/ms℃) |
| Cortical bone | 1300 | 1840 | 0.586 |
| Bone marrow | 1050 | 3552 | 0.5471 |
| Implant (Titanium) | 4500 | 540 | 21.9 |

Reference:

[1] K. Shemtov-Yona, D. Rittel, On the mechanical integrity of retrieved dental implants, Journal of the mechanical behavior of biomedical materials 49 (2015) 290-9.
